# The CO_2_ electrolysing mechanism in single-phase mixed-conducting cathode of solid oxide cell

**DOI:** 10.3389/fchem.2024.1421125

**Published:** 2024-07-19

**Authors:** Zidi Zhu, Yunan Jiang, Lijie Zhang, Hairui Han, Aijun Li, Changrong Xia

**Affiliations:** ^1^ School of Material Science and Engineering, Shanghai University, Shanghai, China; ^2^ Department of Materials Science and Engineering, University of Science and Technology of China, Hefei, China; ^3^ Energy Materials Center, Anhui Estone Materials Technology Co., Ltd., Hefei, China

**Keywords:** solid oxide electrolysis cell, mixed ionic and electronic conducting electrodes, electrochemical impedance spectroscopy, chemical capacitance, numerical simulation

## Abstract

In the field of solid oxide cells (SOC), unveiling the electrochemical reaction and transfer mechanisms in mixed ionic and electronic conducting (MIEC) electrodes is of great importance. Due to the chemical capacitance effects of MIEC materials, SOC often shows large capacitance current during electrochemical tests, which might interfere with the polarization behaviors. This work presents a numerical multiphysical model based on the transport of oxygen species, which accurately and concisely replicates the current-voltage curves of a solid oxide electrolysis cell (SOEC) with MIEC electrodes under various scanning rates. The scanning IV and electrochemical impedance spectra measurement under different SOEC working conditions are combined to enable the separation of Faradic and charging currents. Thus, both the bulk diffusion and surface gaseous diffusion of the oxygen species are encompassed, which explains how the current being generated due to intertwined chemical capacitance effects and chemical reactions in the MIEC electrodes.

## 1 Introduction

Although CO_2_ emission significantly contributes to global warming and climate change, carbon neutralization is gradually becoming the common goal of humanity. Among the cutting-edge technologies to reduce carbon emission, the high-temperature solid oxide electrolysis cell (SOEC) has been proven to be one of the most viable and efficient approach. SOEC technology demonstrates exceptional efficiency in electrolyzing CO_2_ into fuel at elevated temperatures ranging from 650°C to 800°C, achieving energy conversion rates exceeding 90%. Its remarkable stability and prolonged operational lifespan establish it as a pivotal technology for the conversion of CO_2_ into valuable fuels. This capability aligns the SOEC with the imperative need for efficient storage and utilization of renewable energy sources (Li et al., 2023; Qiu et al., 2023). SOEC can reserve renewable electricity by electrolyzing CO_2_ to CO, and CO can be further converted into other useful chemicals and liquid fuels more easily compared with CO_2_ (Leung et al., 2014). Ceria-based materials are widely used as SOEC cathodes due to their high stability and performance under SOEC cathodic conditions. As an efficient electro-catalyst for CO–CO_2_ conversion, doped-ceria usually shows low area-specific polarization resistance (ASR), which is a key parameter to describe the SOEC performance (Nenning et al., 2021; Uecker et al., 2023). Ceria’s high activity in catalyzing redox reactions has been extensively studied in the literature (Zhu and Yu, 2011; Zhang et al., 2012; Sala et al., 2022; Zhang et al., 2024). Nonetheless, SOEC performance can be subject to variability due to fluctuations in temperature and overpotential (Sala et al., 2022; Hauch and Blennow, 2023; Liang et al., 2023). As such, it is necessary to develop a physical model to better understand the mechanisms of mixed ion/electron conduction during the CO_2_ electrolysis process.

Two schools of thought have emerged in the development of SOEC models throughout its history. The first school, rooted in the well-established tradition of classical electrochemical kinetics, focused on the observation that these electrodes tend to conform to Tafel kinetics at moderate-to-high overpotential. However, these results tend to conform to a Butler–Volmer expression, with the exchange current density and anodic and cathodic transfer coefficients obtained at moderate-to-high overpotential. Although some authors concluded that the electrode reaction must be limited by electrochemical kinetics at the interface, the limiting current behavior at high cathodic overpotential suggests otherwise (Gödickemeier et al., 1997; Hu and Liu, 1997; Okamoto et al., 1982).

Nonetheless, gas-diffusion electrodes exhibit multiple rate-determining factors, which change with overpotential or other conditions, so such electrodes deviate substantially from traditional electrochemical–kinetic behavior (Adler et al., 1996; Sala et al., 2022). Therefore, the second school of thought directs attention to the electrode’s impedance and suggests that equivalent RC circuits reveal large capacitances, which exceed the explanation provided by traditional double-layer polarization at the electrode–electrolyte interface (Bauerle, 1969). Kleitz and co-workers studied porous Pt and noble-metal catalysts on YSZ, which responded at significantly lower frequencies than traditional interfacial polarization. These frequencies are outside the scope of traditional interfacial polarization (Schouler et al., 1973). Low-frequency capacitive effects were interpreted by the authors as changes in the concentration of “neutral-O” around the three-phase boundary. Based on their findings, the authors concluded that the overpotential is likely to be a concentration overpotential, at least in part, rather than being attributed solely to electrochemical–kinetic resistance.

Electrochemical impedance spectroscopy (EIS) has emerged as a highly influential experimental technique for analyzing gas-diffusion electrodes in the current solid-state electrochemical literature. EIS measures the response of the current to a sinusoidal voltage modulation with respect to frequency and seeks to identify reaction steps via the timescale (Bauerle, 1969; Bonanos et al., 2018). EIS and current interruption experiments enabled researchers to isolate electrode polarization from the electrolyte on Pt/yttria-stabilized zirconia (YSZ) or Pt/ceria and analyze it as a function of time. However, the analysis of EIS results is still an area under active exploration. The prevalent approach in the scientific literature for fitting impedance spectra involves a connected ensemble of resistors, capacitors, and/or constant phase elements. Although this model is capable of accurately representing the characteristics of various impedance spectra, it often lacks a foundation in the governing physical laws of the system. Instead, it is a pragmatic way of parameterizing the impedance model. Physically motivated models representing equivalent circuits for MIEC have been proposed multiple times in the literature, including the works of Jamnik (2003) and that of Lai and Haile (2005), which is less general but more comprehensive. The Adler–Lane–Steele model is a mathematical model that was developed by Adler et al. (1996). Subsequently, the model has undergone substantial development and has become known as the equivalent circuit model. This model is primarily utilized for describing the kinetics of mixed conducting cathodes, and thus, it warrants careful consideration (Adler, 1998; Flura et al., 2016).

In this paper, a three-electrode system with YSZ as the electrolyte and samarium-doped cerium oxide (SDC) as the working electrode was prepared, with Pt as the counter electrode. We measured the EIS under varying overpotentials. The equivalent circuit model was used to identify chemical capacitance, and a 3D multiphysics model was established by bypassing the Butler–Volmer equation. The Nernst equation and electrochemical reaction were applied along with the detailed mass transfer process that considers chemical capacitance by accounting for gas diffusion and solid diffusion inside the electrodes. With a view toward chemical capacitance, reproducible CV curves were obtained under different scanning speeds.

## 2 Materials and methods

### 2.1 Experiment setup

**FIGURE 1:**
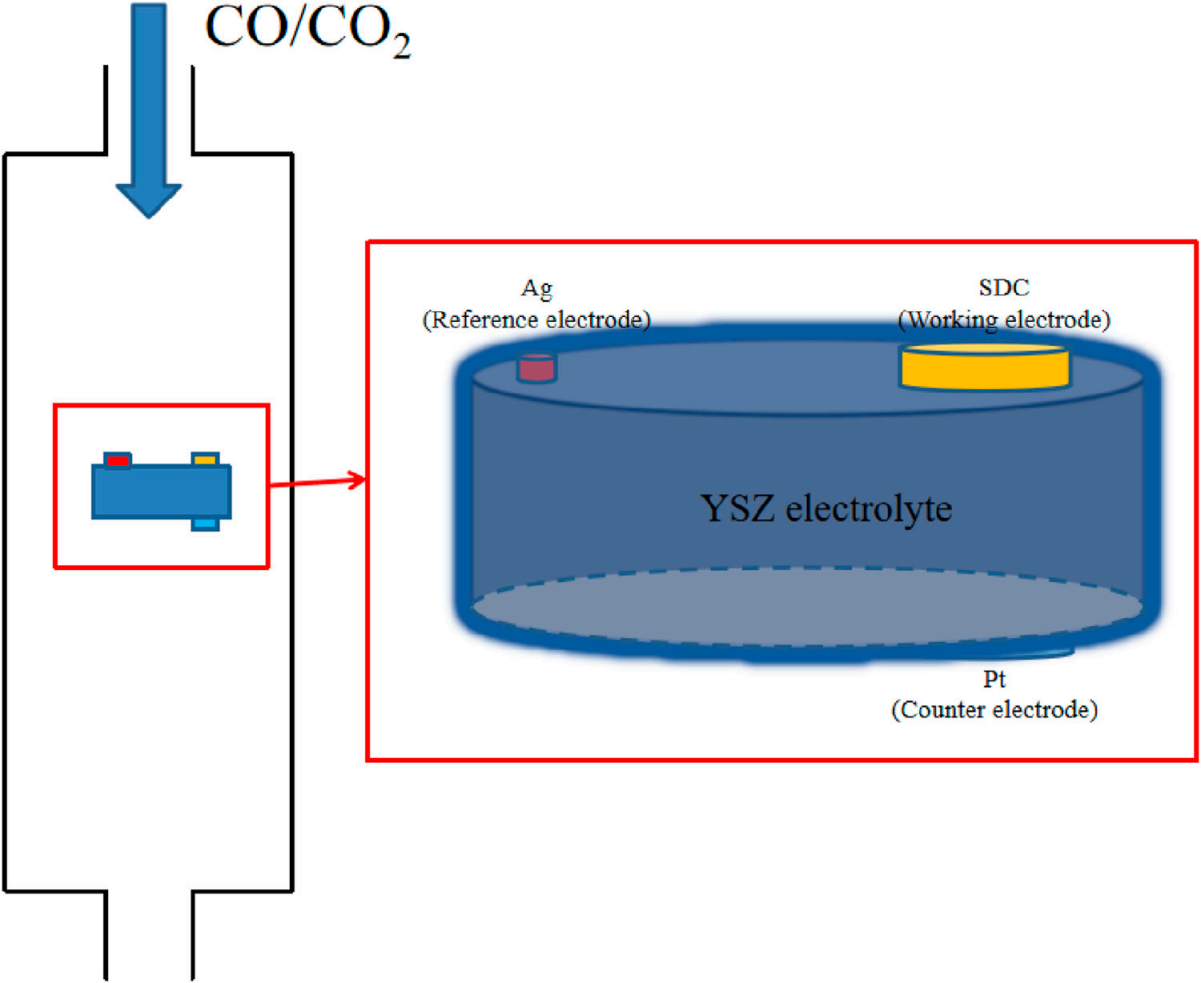
Schematic representation of the three-electrode system. SDC is used as the working electrode (cathode), Pt as the counter electrode (anode), and Ag as the reference electrode.

The resistance of cathodes, anodes, and electrolytes determines the overpotential distribution. In addition, conductivities of these parts are often used to determine overpotential on a single electrode or electrolyte, but this approach can be empirical and problematic for separating the conductivity of individual electrodes. A three-electrode system is implemented to physically determine the exact overpotential on the cathode, as seen in Figure 1.

YSZ powder is ground, pressed, and sintered at 1,200°C to prepare the dense electrolyte, and the thickness and diameter of the sintered YSZ pellet are 0.5 mm and 11 mm, respectively. For the electrochemistry test in the three-electrode system, it is required that the distance separating the working electrode and the reference electrode be no less than three times the thickness of the electrolyte. The cathode material, SDC, is prepared using the glycine-nitrate combustion process, as reported in previous work (Xiang et al., 2014). The desired SDC powder is mixed with alcohol and the dispersant to prepare the SDC suspension, which is ultrasonically sprayed onto one side of YSZ to form a porous cathode with a thickness of approximately 25 μm. Platinum paste is brushed on the opposite side on the YSZ to serve as the counter electrode. Finally, an Ag reference electrode is placed on the slide of YSZ. The areas of the working and counter electrode are 0.2376 cm^2^. Electrochemical characterizations were performed at 750°C with CO/CO_2_ mixed gas of 50% of each, with inlet rates of 30 mL/min.

### 2.2 Reaction and transportation mechanism in MIEC

MIEC presents a favorable option for enhancing electrode performance. In a MIEC SOEC system, the generated electric current can be attributed to either the reduction reaction of CO_2_ or the variation in the concentration of oxygen anions. This relationship can be mathematically described by the following equation:

$$j_{ion}=j_{react}+2F\epsilon_{MIEC}\frac{dc_{ion}}{dt}.$$ (1)

Here, $j_{\text{ion}}$ is the ion current density, $j_{\text{ion}}$ is the reaction current density, $\epsilon_{\text{MIEC}}$ is the volume fraction of MIEC electrodes, $c_{\text{ion}}$ is the concentration of ion, t is time, and F is the Faraday constant.

The most common form of the carbon dioxide reduction reaction is represented by the following equation:

$${\text{CO}}_{2}+{2e}^{-}\to \text{CO}+O^{2-}.$$ (2)

The rate of CO_2_ reduction is primarily determined by the local overpotential. Additionally, the local overpotential will cause variations in the non-stoichiometry of oxygen within the MIEC. It must be stressed that these variations in local overpotential will lead to the variation in the current density with respect to the distance from the surface of the electrode (Nenning et al., 2020).

Eqs 3–7 elucidate the impact of chemical capacitance on the reaction kinetics. For the reduction reaction of CO_2,_ j_react_, the reaction current density is negative, and it can be expressed as follows:

$$j_{react}=j_{surf}*A_{spec},$$ (3)

where $A_{spec}$ is the surface area per unit volume and j_surf_ is the current density of the reaction involving oxide ions at the surface of the MIEC.

The second term in Equation 1 can be equivalently written as the capacitive current density, j_cap_.

$$j_{cap}=2F\epsilon_{MIEC}\frac{dc_{ion}}{dt}=2F\epsilon_{MIEC}\frac{dc_{ion}}{d\eta }*\frac{d\eta }{dt}.$$ (4)

The electrical current is directly related to the rate at which the overpotential changes. This relationship is indicative of a capacitive current. Therefore, it is possible to introduce the concept of chemical capacitance C_chem_, which measures how the concentration of oxygen anions in the MIEC depends on the overpotential. This chemical capacitance can be expressed as follows (Chueh and Haile, 2009):

$$C_{chem}=2F\frac{dc_{ion}}{d\eta }.$$ (5)

By utilizing the chemical capacitance, the capacitive current density can be formulated as follows:

$$j_{cap}=2F\epsilon_{MIEC}\frac{dc_{ion}}{d\eta }*\frac{d\eta }{dt}.$$ (6)

Consequently, the total current generated can be written as follows:

$$\frac{dj_{ion}}{dy}=j_{surf}*A_{spec}+2F\epsilon_{MIEC}\frac{dc_{ion}}{d\eta }*\frac{d\eta }{dt}.$$ (7)

### 2.3 Multiphysics simulation

**FIGURE 2:**
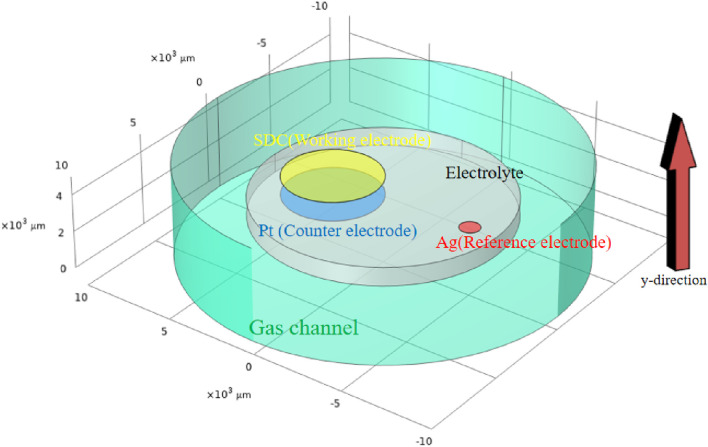
3D model applied in the multi-physical model, with the area and height exactly as in the real situation. The *Y*-direction is designated as the local distance measured from the electrolyte to the electrode surface.

A 3D multi-physics model for SDC-YSZ is developed while fully considering the electrochemical reactions, chemical reactions, ion/electron conduction, and mass/momentum transportation. The schematic representation of the SDC-YSZ SOEC system used in the multi-physics model is shown in Figure 2. The electrode has a surface area of 0.2 cm^2^. Its electrolyte and cathode have thicknesses of 500 µm and 25 μm, respectively.

In operation, CO_2_ is introduced into the cathode. Gaseous CO_2_ molecules diffuse into the porous electrode, where adsorption occurs simultaneously with diffusion. CO_2_ then dissociates with oxygen vacancy on the surface, forming into CO and lattice oxygen. The lattice oxygen will be transported to anodes and get oxidized. As the electrode is a MIEC, dissociation can occur at both the triple-phase and two-phase boundary sites.

The governing equation in the gas channel is expressed in Eq. 8:

$$\frac{\partial c_{i}}{\partial t}-D_{i}\nabla^{2}c_{i}+u\cdot \nabla c_{i}=0.$$ (8)

The governing equation in the porous electrode is expressed in Eq. 9:

$$\frac{\partial \left(\varepsilon c_{i}\right)}{\partial t}-D_{i}^{eff}\nabla^{2}c_{i}=R_{i}+S_{i},$$ (9)

where c_i_ is the concentration of the reactant; *D*_i_ and $D_{i}^{eff}$ are diffusion coefficients of species in the gas and electrodes, respectively; and ***u*** is the velocity field in the gas channel; in the electrode, the velocity field is neglected. $R_{i}$ and $S_{i}$ denote the volume and surface reaction, respectively.

The diffusion coefficient of lattice oxygen is determined as 7.59 ∗ 10^−9^ m^2^/s from the reference (Endler-Schuck et al., 2015).

The mechanism of electrolyzing CO_2_ for SDC as a cathode is concluded in Eqs 10 and 11:

$${\text{CO}}_{2\left(gas\right)}↔{\text{CO}}_{2\left(ad\right)}\;\left(\text{Adsorption}\right),$$ (10)

$${\text{CO}}_{2\left(ad\right)}+{4e}^{-}+V_{O\left(s\right)}^{\cdot \cdot }↔{\text{CO}}_{\left(gas\right)}+O_{O\left(s\right)}^{\times }\left(\text{Dissociation}\right),$$ (11)

where dissociation is the determination step (Feng et al., 2015).

In many articles, the classical Butler–Volmer equation has been applied to determine the reaction current densities. However, some articles argue that the capacitance effect on the electrodes cannot be neglected, and the Tafel method does not apply. Hence, the Butler–Volmer equation may not apply as well. To avoid the problem, assuming that CO will not accumulate at the surface of the electrodes and that the reverse reaction is neglected, the reaction rate is determined by bypassing the Butler–Volmer equation, as demonstrated in Eq. 12.

$$r=k*c\left({\text{CO}}_{2\left(ad\right)}\right)*c{\left(e^{-}\right)}^{4}*c\left(V_{O\left(s\right)}^{\cdot \cdot }\right),$$ (12)

where k is the surface exchange coefficient. It is determined based on the results in Bouwmeester et al. (1994); Adler et al. (1996) as $7*10^{-18}$ mol^16^ mol^−5^ s^−1^ at 750°C with the concentration of electron and oxygen vacancy considered. As ${\text{CO}}_{2\left(ad\right)}$ gets conducted, the lattice oxygen will generate at the same rate.

In this step, electrons are provided by Ce. Under the of CO_2_/CO mixture atmosphere, Ce^4+^ can be reduced to Ce^3+^, leading to more $e^{-}$ and, hence, lower $R_{eon}$ . The concentraion of $c\left(e^{-}\right)$ is determined by the Nernst–Einstein equation, as demonstrated in Eq. 13.

$$n=\frac{\sigma_{eff}\text{KT}}{Dq^{2}},$$ (13)

where K is the Boltzmann constant, T is the absolute temperature, $\sigma_{eff}$ is the effective conductivity of ${Sm}_{0.2}{Ce}_{0.8}O_{2-\delta }$ as 5.88 ∗ 10^−2^ S cm^−1^ at 750°C to determine an initial value of $c\left(e^{-}\right)$ (JU et al., 2014), and D is the coefficient of diffusion of $c\left(e^{-}\right)$ . As mentioned previously, $c\left(e^{-}\right)$ will increase with potential. The determined results of the concentration of $c\left(e^{-}\right)$ are also consistent with the results in DAN et al. (2008).

$V_{O\left(s\right)}^{\cdot \cdot }$ is determined through the ways given below; first, according to the Nernst equation, as demonstrated in Eq. 14.

$$E=E^{⊖}-\frac{RT}{4F}\ln\;\left(\frac{P_{{O_{2}}_{Cat}}}{P_{{O_{2}}_{And}}}\right),$$ (14)

where $P_{{O_{2}}_{Cat}}$ and $P_{{O_{2}}_{And}}$ are partial pressure values of oxygen at the cathode and anode, respectively, and $E^{⊖}$ is the standard cell potential under standard conditions.

In addition, according to the reference, at 750°C, the relation between non-stoichiometric and partial pressure of oxygen is delineated by Eq. 15 (Chueh and Haile, 2009):

$$\ln\delta =-0.25\ln P_{O_{2}}-6.6437.$$ (15)

The partial pressure of oxygen can be determined under different overpotentials, and the non-stoichiometric pressure can be determined with the partial pressure of oxygen. Hence, the concentration of oxygen vacancy $V_{O\left(s\right)}^{\cdot \cdot }$ is determined.

It is noteworthy that the transmission line type has revealed that the overpotential along the *y* direction is not constant but coordinate-dependent. Its relation is derived in Nenning et al. (2014) as Eq. 16:

$$\eta \left(y\right)=U_{0}\frac{\cosh\;\left(y\right)}{\cosh\;\left(L\right)},$$ (16)

where the variable “y” represents the position along the electrode, with 0 indicating the top of the electrode and L indicating the interface with the electrolyte. Moreover, U_0_ denotes the total overpotential experienced across the electrode.

The reaction current and capacitive current, as shown in Eq. 7, are determined by the following equations:

$$I_{react}=F*k*c\left({\text{CO}}_{2\left(ad\right)}\right)*c{\left(e^{-}\right)}^{4}*c\left(V_{O\left(s\right)}^{\cdot \cdot }\right),$$ (17)

$$I_{cap}=-\frac{E-\frac{2*c_{O_{Lat}}*F}{C_{chem}}}{R_{ohm}*{vol}_{eff}},$$ (18)

where $c_{O_{Lat}}$ is the concentration of lattice oxygen, $R_{ohm}$ is the ohmic resistance, and ${Vol}_{eff}$ is the effective volume of the electrode. The effective volume of the electrode is defined by Eq. 19:

$${vol}_{eff}=\epsilon_{SDC}*{vol}_{total},$$ (19)

where $\epsilon_{\text{SDC}}$ is the volume fraction of the SDC electrodes and ${\text{vol}}_{\text{total}}$ is the total volume of the electrode.

Hence, the transportation process of charges inside the electrode is linked with the porosity of the electrode. The total captured current is determined by adding the reaction current and the charging current, as delineated in Eq. 20. It is notable that for negative-direction scanning, the electrode is charging and the captured current is smaller than the reaction current and *vice versa* for positive-direction scanning.

$$I_{total}=F*k*c\left({\text{CO}}_{2\left(ad\right)}\right)*c{\left(e^{-}\right)}^{4}*c\left(V_{O\left(s\right)}^{\cdot \cdot }\right)-\frac{E-\frac{2*c_{O_{Lat}}*F}{C_{chem}}}{R_{ohm}*{vol}_{eff}}.$$ (20)

The overpotential *E* is defined as $E=E_{0}+v_{\text{scan}}*t$ , where $E_{0}$ is the initial overpotential (typically 0 V), $v_{\text{scan}}$ is the scan rate, and t denotes time.

It is imperative to emphasize that the parameters in the multi-physical model are acquired through the EIS method at various overpotentials. Consequently, as far as we know, this is the first time that the two most crucial electrochemical experimental methods of the SOEC cathode are linked, bridging together the two schools of thought for analyzing the cathode of SOECs.

## 3 Results and discussion

### 3.1 EIS results

**FIGURE 3:**
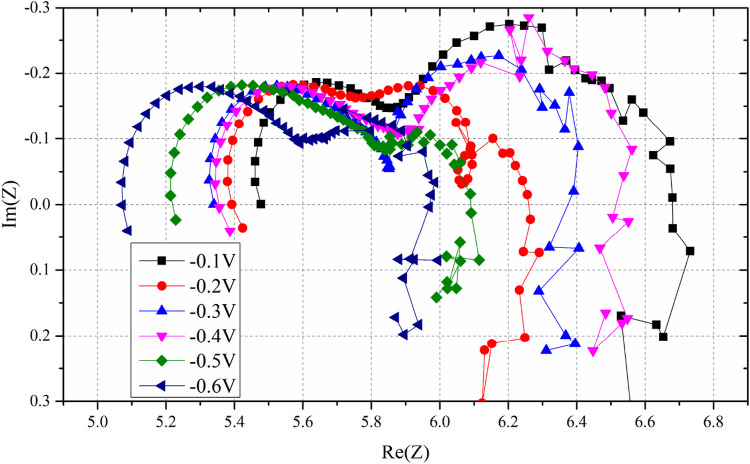
EIS results of overpotential from −0.1V to −0.6V; it is noteworthy that the ohmic resistance decreases with decreasing overpotential.

EIS results of the three-electrode system at different overpotentials are presented in Figure 3. The ohmic, polarization, and total resistances obtained are summarized in Table 1.

**TABLE 1:** Ohmic, polarization, and total resistances at different overpotentials.

Overpotential(V)	Ohmic resistance(Ω)	Total resistance(Ω)	Polarization resistance(Ω)
0	5.55	6.99	1.44
−0.1	5.50	6.68	1.18
−0.2	5.39	6.25	0.87
−0.3	5.34	6.37	1.03
−0.4	5.30	6.46	1.16
−0.5	5.22	6.10	0.89
−0.6	5.07	5.97	0.90

### 3.2 Equivalent circuit models

**FIGURE 4:**
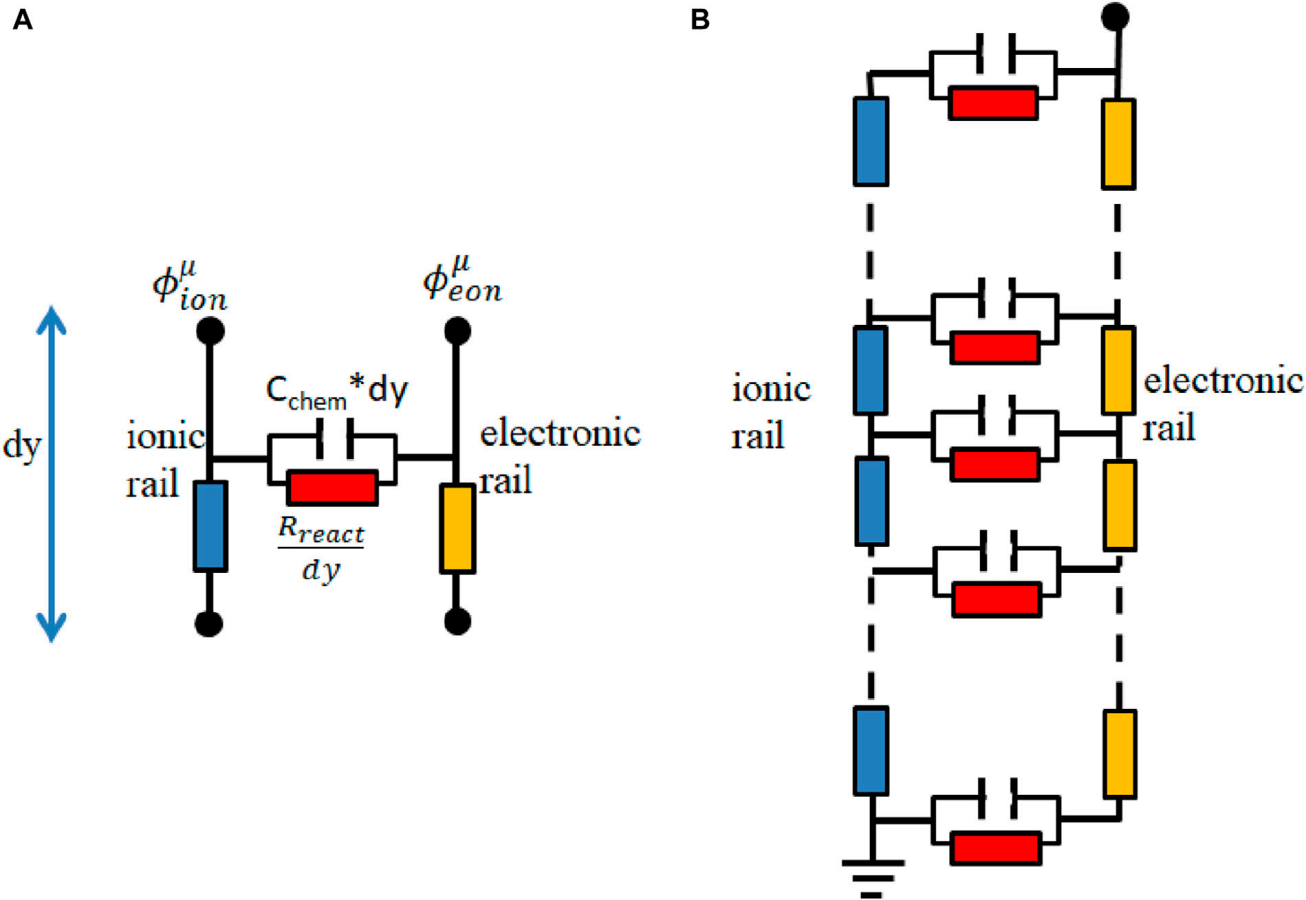
**(A)** Thin film of the electrode with infinitesimal thickness (dy); **(B)** equivalent circuit model.

In this paper, to better understand the process in the single-phase MIEC of SDC, an equivalent circuit model is applied. It consists of differentially thin electrode slices connected in a transmission-line-type circuit. Figure 4 depicts the model.

The circuit model comprises two rails serving as a representation of ion and electron conduction. The differences in voltage present at the rails correspond to the potentials of ions and electrons and are influenced by the spatial distance along the *y*-direction, and y is the local distance to the electrolyte.

R_ion_, R_eon_, R_react_, and C_chem,eff_ are ion conduction resistance, electron conduction resistance, reaction resistance, and effective chemical capacitance of the porous electrode, respectively. The electrochemical reactions connect the ion and electron currents and are symbolized by the horizontal elements, R_react_ and C_chem,eff_.

The impedance of the equivalent circuit model has been mathematically deducted by Bisquert et al. (1999), although the resulting equation is rather complex.

$$Z=\frac{R_{ion}R_{eon}}{R_{ion}+R_{eon}}\left(L+\frac{2\lambda }{\sinh\left(\frac{L}{\lambda }\right)}\right)+\lambda \frac{\left({R_{ion}}^{2}+{R_{eon}}^{2}\right)}{R_{ion}+R_{eon}}\coth\left(\frac{L}{\lambda }\right),$$ (21)

where

$$\lambda =\sqrt{\frac{1}{\left(\frac{1}{R_{react}}+i\omega C_{chem,eff}\right)\left(R_{ion}+R_{eon}\right)}}.$$ (22)

**FIGURE 5:**
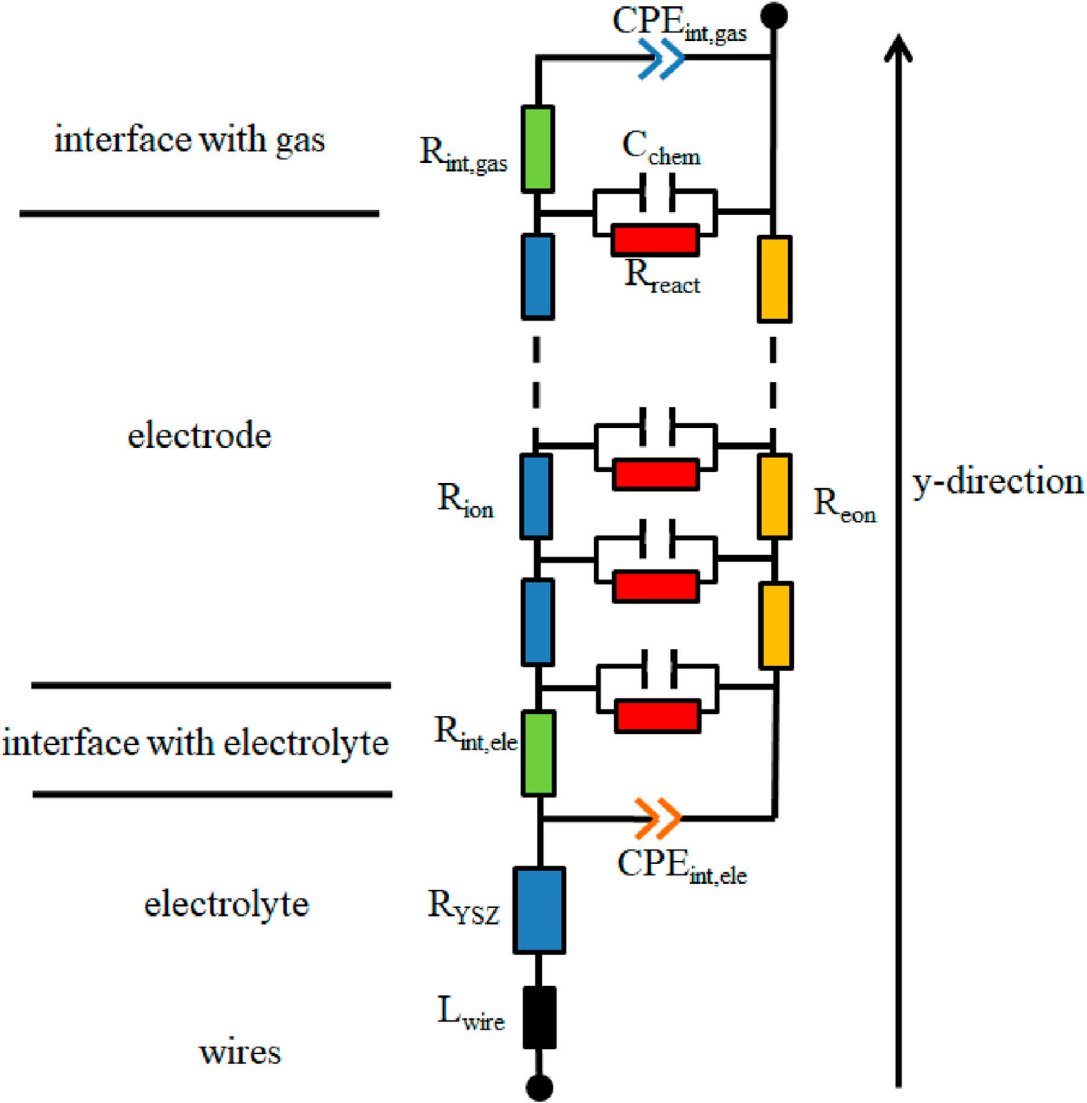
Circuit model used to fit impedance spectra.

Figure 5 presents the resultant circuit utilized for fitting the data. In Figure 5, L_wire_ and R_YSZ_ represent wire inductance and the electrolyte resistance, respectively. CPE_int,ele_ and CPE_int,gas_, as constant phase elements, represent interfacial capacitance between the electrodes and the electrolyte and that between the electrodes and the gas, respectively. Moreover, the circuit also incorporates R_int,ele_ and R_int,gas_, representing interfacial resistance between the electrodes and the electrolyte and that between the electrodes and the gas, respectively (Nenning et al., 2020).

The effective ASR of the electrode arc is articulated in Eq. 23

$${ASR}_{eff}=R_{\mathrm{int},ele}+R_{\mathrm{int},gas}+\frac{R_{ion}R_{eon}}{R_{ion}+R_{eon}}\left(L+\frac{2\lambda }{\sinh\left(\frac{L}{\lambda }\right)}\right)+\lambda \frac{\left({R_{ion}}^{2}+{R_{eon}}^{2}\right)}{R_{ion}+R_{eon}}\coth\left(\frac{L}{\lambda }\right).$$ (23)

**FIGURE 6:**
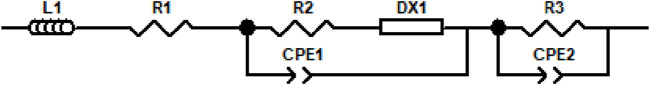
Circuit model applied in Z-View.

Figure 6 illustrates the mathematically identical circuit model used in Z-View software, and Table 2 shows the relationship between the fitting parameters in Z-View.

**TABLE 2:** Relationship between the fitting parameters of Z-View and the parameters of Equation 17.

Parameter of Z-View	Parameter of Equation 21	Unit
DX	None	None; select “Bisquert 2”
DX-R	$R_{eon}$	Ωcm
DX-T	None	F/cm
DX-P	None	1
DX-U	$R_{ion}$	Ωcm
DX-A	None	F/cm
DX-B	None	1
DX-C	$R_{react}$	Ωcm^3^
DX-D	$C_{chem,eff}$	F/cm^3^
DX-E	None	1
DX-F	L	cm

The ohmic resistance decreases with increasing overpotential. Ohmic resistance is the sum of the resistance of the cathode, anode, and electrolyte. The resistance of the anode, electrolyte, and ionic resistance of the cathode are considered constant (Wu et al., 2014). This finding supports the circuit model, which suggested that $R_{eon}$ would decrease with greater Ce conduction.

By employing Z-View software to analyze the transmission-line-type equivalent circuit, crucial parameters such as $R_{eon}$ , $R_{ion}$ , and, notably, $C_{chem,eff}$ can be determined. To validate our model, the obtained value of $C_{chem,eff}$ is input into a multiphysics model to calculate the capacitance current. By combining the capacitance current with the reaction current, the total scanning IV curve is generated, which can then be compared with experimental data to ensure the accuracy of our model.

**FIGURE 7:**
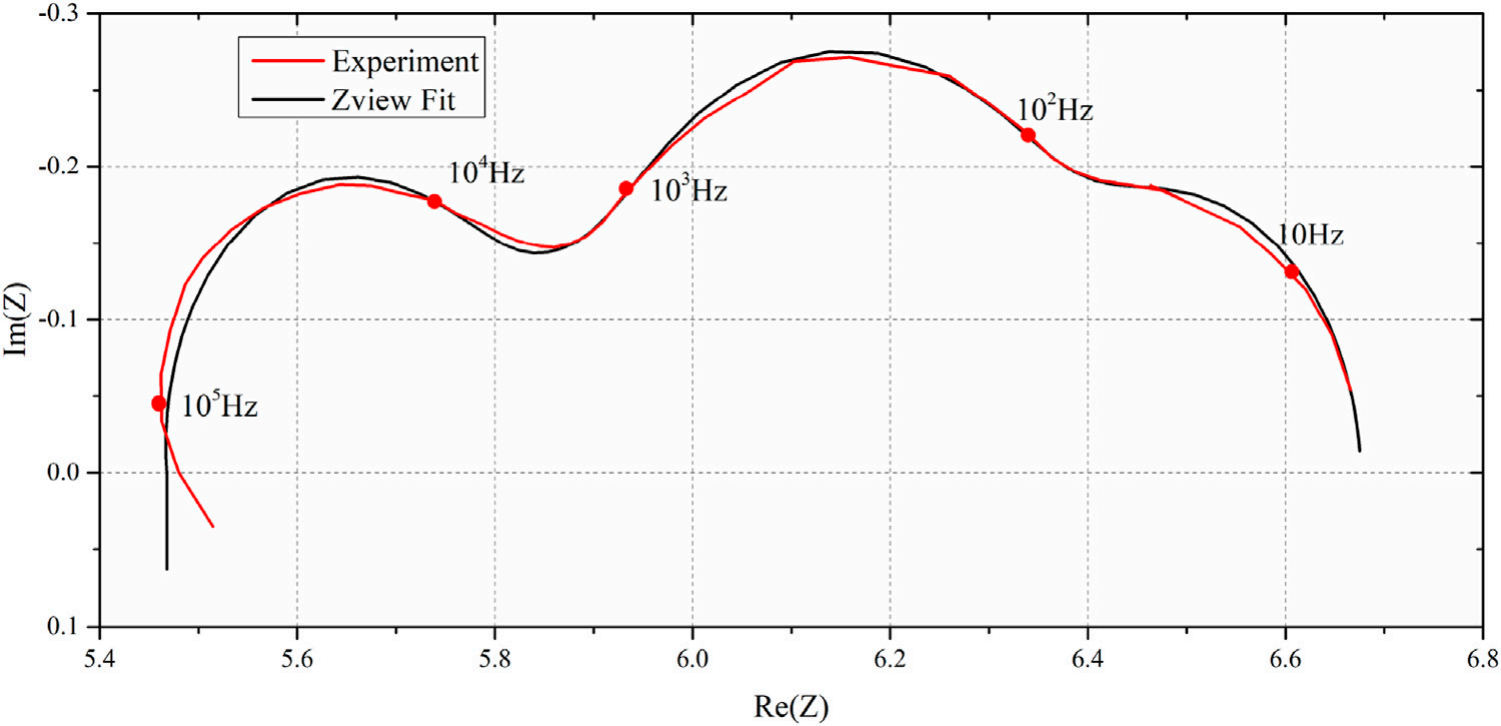
Experiment and Z-View fit results of EIS at an overpotential of −0.1 V.

As an example of fitting the equivalent circuit model, the EIS at −0.1 V is used. Table 3 shows the obtained parameters. Notably, the parameter names in Table 1 and Table 3 remain consistent. For instance, “DX1-R” in Table 3 corresponds to “DX-R” in Table 1, as specified in Equations 21, 22. Additionally, the “1” in “DX1-R” signifies its representation of values in the DX1 element in Figure 6. Figure 7 shows experiment and Z-View fit results of EIS at an overpotential of −0.1 V. The obtained chemical resistance (DX-D) shows consistency with the results reported in Chueh and Haile (2009). The maximum error in the impedance module is 0.9% at the same frequency, while the error in the imaginary part is 7.89% at the same frequency.

**TABLE 3:** Parameters applied in Z-View at an overpotential of −0.1 V.

Parameter of Z-View	Parameter of Equation 21
L1	1E-7
1	5.468
R2	0.255
DX1	Select “Bisquert 2”
DX1-R	17,000
DX1-T	0
DX1-P	1
DX1-U	1,800
DX1-A	0
DX1-B	1
DX1-C	1.55E-5
DX1-D	32,000
DX1-E	1
DX1-F	3.37E-5
CPE1-T	0.0123
CPE1-P	1.07
R3	0.295
CPE2-T	21
CPE2-P	0.97

The most critical parameter, $C_{chem,eff}$ , which is applied in the multiphysics model, is defined by fitting the values of $C_{chem,eff}$ at various overpotentials by $C_{chem,eff}=30000*e^{\frac{0.06*F*E}{\text{RT}}}$ .

### 3.3 IV results

**FIGURE 8:**
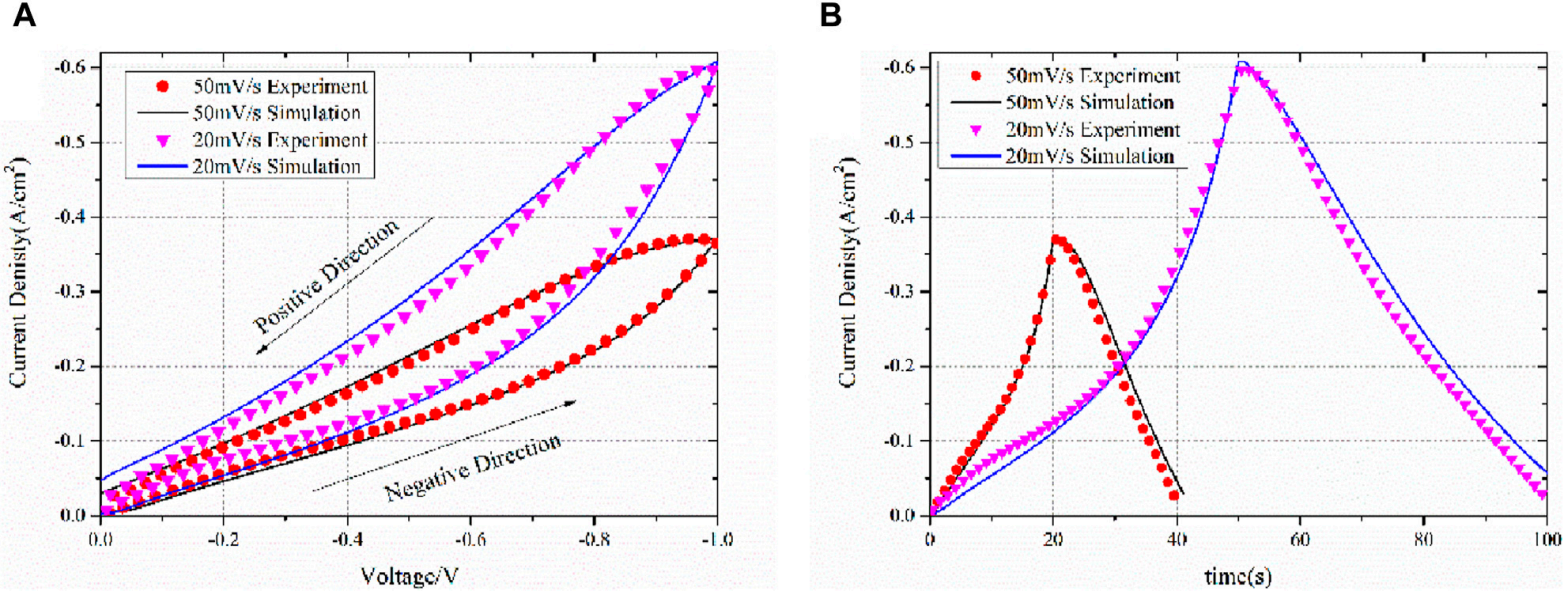
Experiment and simulation IV results of scanning rates of 50 mV/s and 20 mV/s with respect to **(A)** voltage and **(B)** time.

The IV curves are obtained for different scanning rates, including 50 mV/s and 20 mV/s. The obtained IV curves are compared with the simulation results shown in Figure 8 with respect to (a) voltage and (b) time. The IV curves were first obtained in the negative direction and then immediately turned to the positive direction using the same scanning rate.

The simulated IV curves are consistent with the experimental results, thus confirming the validity of the equivalent circuit model theory. It is important to reiterate that the model does not employ the Butler–Volmer equation. Determining the kinetic parameters of the Butler–Volmer equation proves challenging. For numerous models, the exchange current density is derived from assumptions, and the transfer coefficient is typically assumed to be 0.5 for both anodes and cathodes (Xu et al., 2018; Yang et al., 2018; Wang et al., 2020). However, in the case of several electrodes, particularly porous ones, the transfer coefficient deviates from 0.5 due to the influence of the electrode’s topology (Yan et al., 2015).

As shown in Figure 8, after changing the scanning direction, the current exhibits a slight increase followed by a decrease. This phenomenon is well-reproduced in simulations, even at different scanning rates. As previously indicated, the chemical capacitance strongly affects the current. During the negative scanning direction, the capacitance will be charged, resulting in a smaller current than normal. During the positive scanning direction, the capacitance will be discharged, causing a larger than normal current. Following the reversal of the scanning direction, the electrode is fully charged as a body capacitance, and the amount of charged particles is quite large. This results in a very large discharge current, which can even be greater than the reaction current, making the current greater than the peak of the negative direction scanning. This phenomenon is, however, unsustainable, and the current subsequently drops as the overpotential scan proceeds in the positive direction.

The current density at IV is higher for a scanning rate of 20 mV/s than for a scanning rate of 50 mV/s, and this is due to the chemical capacitance. At lower scanning rates, charge has more time to charge the capacitance. At the end of the negative scanning at 20 mV/s, the charge amount in the whole electrode is larger than that at 50 mV/s. According to Equation 28, the captured current density is higher at 20 mV/s than at 50 mV/s. This phenomenon has been reported in previous studies, such as Jacobsen et al. (2001).

It is crucial to acknowledge that while electrochemical impedance spectroscopy (EIS) was conducted up to −0.6 V, the current–voltage (IV) curve simulation extended to −1.0 V. Conducting EIS at high overpotentials, such as −1.0 V, poses a potential risk of damaging the electrode, especially in a three-electrode system where the overall cell potential can exceed −2 V. Given that the normal operating potential of the SDC working electrode is lower, EIS data collected within the range of 0 V to −0.6 V are adequate to establish the relationship between impedance and overpotential. However, the scanning IV curve enables a broader potential range to be explored rapidly, with a minimal risk of electrode damage. This wider potential range covered by the IV curve simulation further validates the robustness of our model and offers valuable insights into the electrochemical behavior across a broader operating range.

**FIGURE 9:**
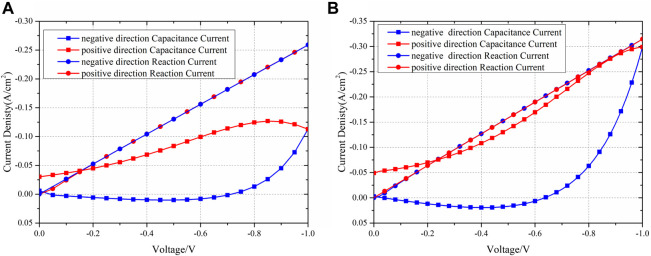
Experiment and simulation IV results of the separated reaction and capacitance currents of scanning rates of **(A)** 50 mV/s and **(B)** 20 mV/s.

One of the primary advantages of simulation is its ability to effectively separate each physical process. Figure 9 presents the current contributed by the reaction and chemical capacitance. The chemical reaction remains nearly identical, regardless of the scanning direction. However, the current density due to the chemical capacitance significantly varies depending on the scanning direction. The maximum difference in chemical capacitance can reach 72.15% of the reaction current at the same overpotential. The concept of chemical capacitance can also elucidate the phenomenon where the current is zero at the beginning but not zero when scanning back to zero overpotential. This occurs because, at the start, there is no charged lattice oxygen. However, when scanning back to zero overpotential, the discharge process is slower than the reduction of overpotential, and thus, the charged lattice oxygen remains in the entire electrode, leading to a non-zero capacitance current. Consequently, even though the reaction current becomes zero, the current associated with chemical capacitance persists. At this point, the total current primarily consists of the chemical capacitance current.

It is important to note that the capacitive current is higher at a scan rate of 20 mV/s compared to that at 50 mV/s at −1 V. This difference can be attributed to the calculation of the capacitive current using Equation 18, where at a certain overpotential, all parameters except $c_{O_{Lat}}$ remain constant. As $c_{O_{Lat}}$ increases, the numerator in Equation 18 decreases due to its negative sign, resulting in an increase in $I_{cap}$ . At lower scanning rates, there is more time for oxygen to oxidize and form $c_{O_{Lat}}$ , leading to a larger $c_{O_{Lat}}$ value. Consequently, the capacitive current is higher at 20 mV/s compared to 50 mV/s at −1 V.

Thus, it can be concluded that the difference in current observed during the scanning process in different directions is primarily caused by the chemical capacitance. Additionally, the various current densities at different scanning rates are also attributable to the chemical capacitance.

## 4 Conclusion

The study employed a three-electrode system to determine the precise overpotential on the SDC, and the corresponding EIS and scanning IV were measured. The transmission-line-type model was adopted to determine the kinetic parameters of a single-phase MIEC. To verify the validity of the calculated parameters, a multi-physical model based on the transport of oxygen species was established and the current–voltage curves of SOECs with MIEC electrodes under various scanning rates were accurately and concisely replicated. Our study of the single-phase SDC cathodes link the EIS method and scanning IV together for the first time, leading us to conclude that chemical capacitance significantly affects the reaction and transportation processes of the SOEC’s MIEC, and the Butler–Volmer equation may not always be necessary to describe MIEC behavior. Furthermore, for the first time, the separation of Faradaic and charging currents on SOEC cathodes was achieved.

## Data Availability

The original contributions presented in the study are included in the article/Supplementary Material; further inquiries can be directed to the corresponding author.
